# Safety and feasibility of fasting in combination with platinum-based chemotherapy

**DOI:** 10.1186/s12885-016-2370-6

**Published:** 2016-06-10

**Authors:** Tanya B. Dorff, Susan Groshen, Agustin Garcia, Manali Shah, Denice Tsao-Wei, Huyen Pham, Chia-Wei Cheng, Sebastian Brandhorst, Pinchas Cohen, Min Wei, Valter Longo, David I. Quinn

**Affiliations:** USC Keck School of Medicine, Norris Comprehensive Cancer Center, 1441 Eastlake Ave. #3440, Los Angeles, CA 90033 USA; Department of Preventive Medicine, USC Keck School of Medicine, 1441 Eastlake Ave, #4427, Los Angeles, 90033 CA United States; USC Keck School of Medicine, Department of Obstetrics and Gynecology, 1441 Eastlake Ave, #3440, Los Angeles, 90033 CA United States; Longevity Institute, University of Southern California Davis School of Gerontology, Department of Biological Sciences, 3715 McClintock Avenue, Los Angeles, 90089 CA United States

**Keywords:** Fasting, Chemotherapy, Neutropenia, Oxidative stress, Insulin-like growth factor

## Abstract

**Background:**

Short-term starvation prior to chemotherapy administration protects mice against toxicity. We undertook dose-escalation of fasting prior to platinum-based chemotherapy to determine safety and feasibility in cancer patients.

**Methods:**

3 cohorts fasted before chemotherapy for 24, 48 and 72 h (divided as 48 pre-chemo and 24 post-chemo) and recorded all calories consumed. Feasibility was defined as ≥ 3/6 subjects in each cohort consuming ≤ 200 kcal per 24 h during the fast period without excess toxicity. Oxidative stress was evaluated in leukocytes using the COMET assay. Insulin, glucose, ketones, insulin-like growth factor-1 (IGF-1) and IGF binding proteins (IGFBPs) were measured as biomarkers of the fasting state.

**Results:**

The median age of our 20 subjects was 61, and 85 % were women. Feasibility criteria were met. Fasting-related toxicities were limited to ≤ grade 2, most commonly fatigue, headache, and dizziness. The COMET assay indicated reduced DNA damage in leukocytes from subjects who fasted for ≥48 h (*p* = 0.08). There was a non-significant trend toward less grade 3 or 4 neutropenia in the 48 and 72 h cohorts compared to 24 h cohort (*p* = 0.17). IGF-1 levels decreased by 30, 33 and 8 % in the 24, 48 and 72 h fasting cohorts respectively after the first fasting period.

**Conclusion:**

Fasting for 72 h around chemotherapy administration is safe and feasible for cancer patients. Biomarkers such as IGF-1 may facilitate assessment of differences in chemotherapy toxicity in subgroups achieving the physiologic fasting state. An onging randomized trial is studying the effect of 72 h of fasting.

**Trial registration:**

NCT00936364, registered propectively on July 9, 2009.

**Electronic supplementary material:**

The online version of this article (doi:10.1186/s12885-016-2370-6) contains supplementary material, which is available to authorized users.

## Background

Platinum chemotherapy is a mainstay of combination systemic therapy for many solid tumors, with the ability to reduce the risk of cancer recurrence after curative surgery in some situations, or to extend survival in advanced disease. However toxicity frequently limits the amount of chemotherapy that can be administered. Both the efficacy and toxicity of chemotherapy agents, including platinum drugs, are related to oxidative cellular damage. Preclinical studies have shown that the heart, liver, and renal tissue may be protected from toxicity by the concurrent administration of antioxidants [1–3]. The limitation of this approach has been concern over a possible attenuation of efficacy against malignant cells, although this has not been substantiated in the available randomized trial data [4]. A more appealing approach would be to differentially induce protection in normal host cells without reducing, or potentially even increasing, susceptibility of cancer cells to chemotherapy. Cell culture experiments have identified that chemotherapy toxicity to normal primary cells was reduced when cultured in conditions mimicking fasting, while neoplastic cells did not experience the same protection, and in some cases be sensitized to the chemotherapeutic cytotoxicity in the low-glucose and low growth factor environment [5, 6]. Further experiments with xenografts in mice revealed that short-term starvation (STS) for 48 h prior to chemotherapy treatment significantly reduced side effects and death from high-dose chemotherapy when compared to mice fed with standard diets prior to receiving chemotherapy, leading to a hypothesis that fasting induces oxidative stress resistance [5]. The mice subjected to STS regained most of the weight lost during the 4 days after chemotherapy, whereas the control mice lost a significant proportion of their weight in the same post-chemotherapy period, potentially reflecting their experience of chemotherapy toxicities of anorexia and nausea. The overall response of the mice exposed to STS was encouraging for the safety of translating this concept into human cancer patients.

Powerful and wide-ranging metabolic and gene expression changes are induced by calorie restriction in normal cells, including upregulation of antioxidants and DNA repair pathways, in part mediated by dampening the nutrient-sensing and pro-proliferative pathways such as IGF-1/Akt and mTOR [6]. Oncogene expression, affecting the same pro-growth signaling cascades among others, prohibit a fasting-like response in cancer cells which continue to proliferate, and cancer cells may actually be sensitized to toxins in the setting of nutrient deprivation [7]. Studies in healthy volunteers have revealed that within 22 and 48 h of fasting, blood glucose and insulin levels decrease significantly, and blood ketones increase [8, 9]. STS has been shown to induce a 40 % reduction in circulating insulin-like growth factor-1 (IGF-1) as well as changes in IGF-1 binding protein (IGFBP) levels in mice [10, 11]. These changes represent a potential set of biomarkers for identifying when a protective state may occur, although they only represent a subset of the changes induced by fasting.

As a first step in exploring the ability of fasting to induce differential stress resistance in humans, we performed a clinical trial to determine the safety and feasibility of fasting prior to chemotherapy administration in human cancer patients. We sought to identify a recommended fasting duration to be studied in a subsequent randomized trial, embedding correlative studies to generate preliminary data regarding biomarkers of the fasting state and to evaluate oxidative stress in host leukocytes as proof of principle. To assess safety and compliance, we designed a dose-escalation protocol in a “real-world” setting of patients with advanced cancer receiving platinum-based combination chemotherapy.

## Methods

Eligible patients had cancer for which platinum-based combination chemotherapy without concurrent radiation was being recommended with curative (peri-operative) or palliative intent. Because fasting was timed around the administration of platinum, regimens in which platinum was administered consecutively for more than 2 days (ex: Bleomycin, Etoposide, Cisplatin for germ cell tumors) were not eligible. Patients may have begun receiving platinum chemotherapy (1–2 cycles of the chemotherapy could have already been administered), provided at least 2 more cycles were planned during which fasting could occur. Chemotherapy was administered at the treating physician’s discretion; standard antiemetics were administered, including dexamethasone and 5HT3 inhibitors. Subjects were excluded if they had diabetes, low body mass index (<20.5), or had lost more than 10 % of their weight in the preceding year.

**Fig. 1:**
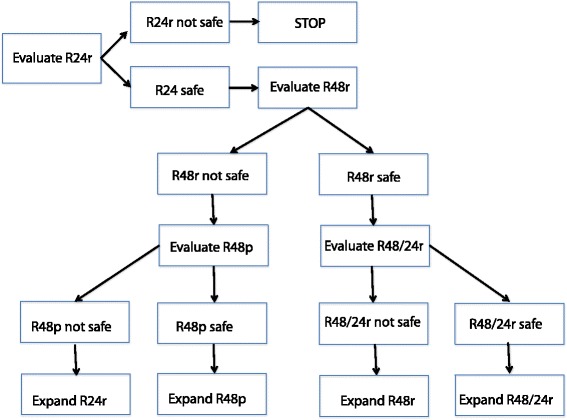
Schema of the trial design; cohorts and strategy for escalating fasting duration. R24r signifies 24 hours of fasting without planned food, only rescue. R48r is 48 hours of fasting prior to chemotherapy, without planned food, only rescue. If safety or feasibility failed in the cohort of 48 hours fasting, we planned a “de-escalation” to a 48 hour fasting period with planned rescue food: R48p. However, if 48 hours fasting was safe and feasible, we would escalate to 72 hours fasting, broken down as 48 hours before chemotherapy and 24 hours after, R48/24r

Escalation of fasting began at a “dose” level of 24 h, and each cohort consisted of 6 subjects; the design is summarized in Fig. 1. Subjects were instructed to consume zero calories, but ample water and non-caloric beverages. However subjects were advised that if they had symptoms related to fasting (such as feeling faint, weak, dizzy, etc.) that they should consume a small amount of juice or food, aiming to stay under 200 kcal in a 24 h period. All food consumed was recorded in a food diary, including quantity, so that calorie intake could be estimated. There were no specific requirements for what to eat on all other days of the chemotherapy cycle, although a transition diet (ex: starting with small quantities of soft cooked foods, then advancing to regular diet) was recommended at the completion of longer fasting durations.

A patient was classified as “compliant” if he or she consumed fewer than 200 kcal/24 h during the fasting period for 2 consecutive courses of chemotherapy. Feasibility for the fasting regimen was defined as 3 or more complaint subjects who did not experience unacceptable fasting-related toxicity. Unacceptable fasting-related toxicity was defined as: patients being hospitalized during the fasting period (for reasons that are not attributed to disease, chemotherapy or post-operative complications) OR patients experiencing any Grade 3+ adverse events not attributed to disease, chemotherapy or post-operative complications during the fasting period. Toxicities were recorded at the start of each chemotherapy cycle, and were graded according to CTCAE v4.0. The safety benchmark was set at 0 subjects in the 6 person cohort experiencing unacceptable toxicity related to fasting.

If the 24 h fasting cohort did not meet the criteria for safety AND feasibility, the protocol would be terminated. If safety and feasibility were met, escalation would proceed to 48 h, and then if the 48 h cohort met the criteria for safety and feasibility, the plan was to escalate to 72 h fasting (48 h before and 24 h after completion of platinum chemotherapy, the split timing based on preclinical observations). If feasibility or safety were not met at 48 h, a 48 h fasting cohort would be opened with a specific low-calorie diet plan. If the 72 h cohort was opened but was found to be not safe or feasible, expansion of the 48 h cohort would occur (see Fig. 1).

After enrollment, baseline levels of glucose, insulin, and IGF-1 pathway markers were measured. Subjects were instructed to begin the fast 24 (or 48) hours before the expected completion of their platinum infusion, thus requiring coordination with the infusion centers and careful estimation of pre-medication and hydration infusion times. Fasting was undertaken prior to chemotherapy during 2 chemotherapy treatment cycles. Subjects were allowed to choose whether to continue fasting or to consume a regular diet prior to subsequent chemotherapy treatments.

**Fig. 2:**
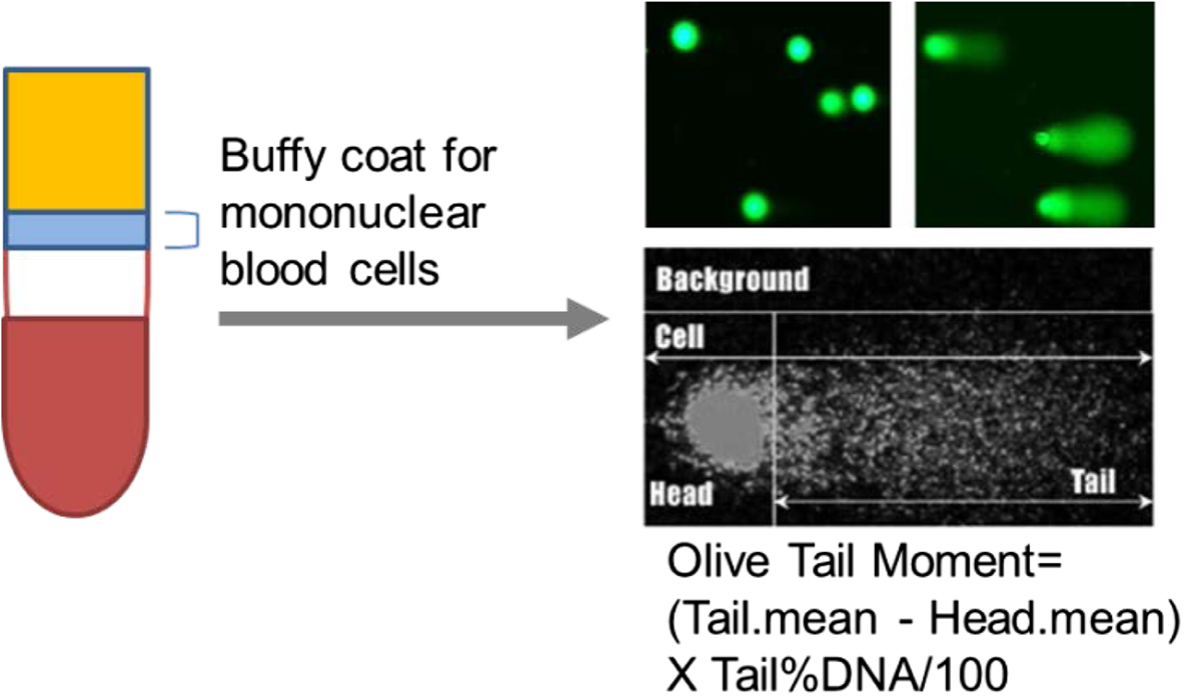
DNA damage in host cells was measured using peripheral blood mononuclear cells from study subjects using the COMET assay

Blood samples were collected after fasting but before administration of premedications and chemotherapy, and then again 24 h after completion of chemotherapy. Serum samples were analyzed for IGF-1 and IGFBP1 by an in-house ELISA developed in the Cohen laboratory [12, 13]. Single cell gel electrophoresis assay (comet assays [14]) for the detection of DNA damage was performed with Trevigen Comet Assay kits according to the manufacturer’s protocols Fig. 2. In brief, cells isolated from buffy coats were embedded in Comet LM Agarose and then lysed followed by electrophoresis in TBE buffer. Cells were stained with SYBR Green and imaged. Analysis was performed using CometScore™, where values of Olive moment were generated [15]. Biomarker data, except the Comet assay, were logarithm-transformed prior to analysis; to compare differences among the 3 fasting cohorts, only “compliant” patients were included. Geometric means and associated 95 % confidence intervals were calculated; *p*-values for the biomarkers analyses were based on the F-test from regression analyses with patients classified as a random effect. Categorical data were summarized with numbers and percentages; Fisher’s exact test was used to test association and Mantel-Haenszel test for trend was used for testing for trend. Intent-to-treat analysis was used for clinical endpoints.

## Results

After Institutional Review Board approval, 20 subjects provided written informed consent and were enrolled from October 2009 to November 2012 at the University of Southern California (USC) Norris Comprehensive Cancer Center and the Los Angeles County/USC Medical Center. Trial design schema is provided in Additional file 1: Figure S1. The median age was 61 (range 31–75). A description of the tumor types and chemotherapy regimens and disease states for study participants is presented in Table 1. Demographics and study treatment course are summarized in Table 2. Nine subjects were Hispanic, 2 were African-American, 8 were non-Hispanic white, and 1 patient was Asian. 17 (85 %) were women.

**Table 1:** Tumor types and chemotherapy regimens used by cohort for participants in this study

Cancer type	Chemotherapy regimen	Disease state
24 h cohort		
Urothelial	Gemcitabine + Cisplatin	Metastatic
Urothelial	Gemcitabine + Cisplatin	Adjuvant
NSCLC	Gemcitabine + Cisplatin	Metastatic
Urothelial	Gemcitabine + Cisplatin	Neoadjuvant
Ovarian	Carboplatin + Paclitaxel	Adjuvant
Uterine	Carboplatin + nab-Paclitaxel	Metastatic
48 h cohort		
Ovarian	Carboplatin + Paclitaxel	Adjuvant
Breast	TCH	Adjuvant
Breast	TCH	Adjuvant
Breast	TCH	Adjuvant
Urothelial	Gemcitabine + Cisplatin	Neoadjuvant
Breast	TCH	Adjuvant
Ovarian	Carboplatin + Paclitaxel	Metastatic
72 h cohort		
Ovarian	Carboplatin + Paclitaxel	Metastatic
Urothelial^a^	Gemcitabine + Cisplatin	Neoadjuvant
Uterine	Carboplatin + Paclitaxel	Adjuvant
Breast	TCH	Neoadjuvant
Urothelial	Gemcitabine + Cisplatin	Neoadjuvant
Ovarian	Carboplatin + Paclitaxel	Adjuvant
Ovarian	Carboplatin + Paclitaxel	Metastatic

*TCH* Docetaxel, carboplatin, trastuzumab

^a^This patient was ineligible due to BMI 20.1 (original protocol required ≥20.5 but the protocol was subsequently amended to allow patients with normal BMI ≥18.5 given a lack of weight loss observed in the first 2 cohorts) and the data were included from 2 successful fasting cycles

**Table 2:** Baseline clinical and demographic characteristics of the study cohort, as well as compliance and radiographic response (RECIST) to chemotherapy

Characteristic	Median (range)	Number (%)
Age	61 (31–75)	
Gender	Female	17 (85 %)
	Male	3 (15 %)
Race	Caucasian	8 (40 %)
	Hispanic	9 (45 %)
	Black	2 (10 %)
	Asian	1 (5 %)
ECOG Performance Status	0	12 (60 %)
	1	8 (40 %)
Total Fasting Cycles Completed	1	3 (15 %)
	2	15 (75 %)
	>2	2 (10 %)
Best objective (RECIST) response	CR	2 (10 %)
	PR	6 (30 %)
	SD	3 (15 %)
	PD	1 (5 %)
	N/A0 (adjuvant therapy)	8 (40 %)

*CR* Complete response, *PR* Partial response, *SD*, Stable disease, *PD* Progressive disease

In the 24 h cohort, all 6 subjects were evaluable, but only 4 successfully fasted for 2 chemotherapy cycles; no significant fasting toxicities were noted. Reasons for failure included forgetting (*n* = 1) and desire not to fast due to social constraints with visiting friends/family (*n* = 1). In the 48 h cohort, 6 subjects were evaluable and 5 were compliant; one inevaluable due to change in chemotherapy plans before completing 2 cycles and was replaced. Of the 6, one failed to regain 25 % of lost weight during the rest of the treatment cycle and per protocol could not fast again. In the 72 h cohort, 7 subjects were enrolled but 1 was ineligible due to normal BMI, but lower than eligibility criteria; with subsequent protocol amendment, this patient would have been eligible and is included in the analysis. All subjects in the 72 cohort consumed some calories, but 4 were reported compliance with <200 kcal/24 h of fasting. Overall, 13 of the 20 patients recorded calorie consumption falling within the < 200 kcal/24 h compliant range. Fasting-related symptoms included fatigue (10 subjects, 6 grade 1 and 4 grade 2), grade 1 headache (6 subjects), dizziness (6 subjects), hypoglycemia (3 subjects), grade 1 weight loss (2 subjects), hyponatremia (2 subjects) and hypotension (1 subject); there were no grade 3 or 4 fasting-related toxicities.

Chemotherapy-related toxicities were collected and tabulated, with data summarized in Table 3. Grade 3 or 4 neutropenia occurred in 4 of 6 patients in the 24 h cohort (67 %), 1 of 7 patients in the 48 h cohort (14 %) and 2 of 7 patients in the 72 h cohort (29 %) *p* = 0.17 for 24 h compared to the 48 + 72 h cohort patients. Routine granulocyte colony stimulating factor (GCSF) support was not prohibited, but was not used in our study population except in one patient after neutropenia had occurred. All 6 subjects in the 24 h fasting cohort experienced grade 1 or 2 nausea, compared to 6 of 7 (87 %) in the 48 h cohort and 3 of 7 (43 %) in the 72 h cohort (*p* = 0.019, test for trend); 5/6 (83 %) subjects in the 24 h fasting cohort experienced grade 1 or 2 vomiting, compared to 3/7 (43 %) in the 48 h cohort and none (0 of 7) in the 72 h cohort (*p* = 0.003, test for trend).

**Table 3:** Chemotherapy related toxicities. Rates of selected chemotherapy-related toxicities experienced by patients in the fasting cohorts, with CTC adverse event v4.0 grading. Only the grades for which events occurred are shown

Toxicity		24 h	48 h	72 h
		# (%)	# (%)	# (%)
		*N* = 6	*N* = 7	*N* = 7
Constitutional/General				
Fatigue	Grade 1 or 2	6 (100 %)	5 (71 %)	6 (86 %)
Alopecia	Grade 1	6 (100 %)	5 (71 %)	7 (100 %)
Gastrointestinal				
Nausea	Grade 1 or 2	6 (100 %)	6 (86 %)	3 (43 %)
Vomiting	Grade 1 or 2	5 (83 %)	3 (43 %)	0
Constipation	Grade 1 or 2	3 (50 %)	2 (28 %)	3 (43 %)
Diarrhea	Grade 1 or 2	2 (33 %)	0	4 (57 %)
	Grade 3	0	1 (14 %)	0
Hematologic				
Neutropenia	Grade 1 or 2	1 (17 %)	3 (43 %)	1 (14 %)
	Grade 3 or 4	4 (67 %)	1 (14 %)	2 (29 %)
Thrombocytopenia	Grade 1 or 2	4 (67 %)	1 (14 %)	1 (14 %)
	Grade 3 or 4	0	1 (14 %)	0
Laboratory/Metabolic				
Hyponatremia	Grade 1	1 (17 %)	1 (14 %)	1 (14 %)
	Grade 3	1 (17 %)	0	0
Hypokalemia	Grade 1	1 (17 %)	2 (28 %)	0
Hyperglycemia	Grade 1 or 2	4 (67 %)	1 (14 %)	0
Elevated AST/ALT	Grade 1	4 (67 %)	0	3 (43 %)
Neurologic				
Peripheral Neuropathy	Grade 1	3 (50 %)	1 (14 %)	1 (14 %)
Dizziness	Grade 1 or 2	1 (17 %)	5 (71 %)	2 (29 %)

Pathologic complete responses in the setting of radiographic complete responses were observed in 2 patients (Table 2). Partial radiographic responses were seen in 6 of the 20 patients, of which 1 patient subsequently was found to have pathologic complete response. Three patients had stable disease as their best radiographic response, of which 2 underwent surgery and had pathologic complete response. Four of the pathologic complete responses were seen in the 72 h cohort; the other was in the 48 h cohort. One patient had progressive disease during treatment (48 h cohort), and 6 were not evaluable because they were treated in the adjuvant setting.

Changes in insulin, glucose, IGF-1, IGFBP, and β-hydroxybutyrate levels are summarized in Table 4, with graphical summary of IGF-1 levels in Additional file 1: Figure S1. Among the compliant patients, blood glucose did not change significantly or consistently (*p* = 0.35). In the 24 h cohort, 4 of 6 subjects reported compliance with fasting and recorded <200 kCal consumed; in these, 4 patients, insulin levels decreased by a mean of −56 % just after chemotherapy following the first fast. In the 48 h cohort, in 6 compliant patients, the insulin level decreased by 27 % and in the 7 compliant patients in the 72 h cohort, insulin levels decreased by 42 % at 48 h after fasting. Although patterns were suggestive, given the patient-to-patients variability, the 3 cohorts did not differ significantly in terms of changes over time (*p* = 0.35). Just after chemotherapy following the first fast, IGF-1 levels decreased by a mean of −30 % (−44 %, −12 %) in the 24 h cohort, −33 % in the 48 h cohort, and −8 % in the 72 h cohort (*p* = 0.32 comparing all 3 groups overall the times). After chemotherapy following fasting, the serum beta-hydroxybutyrate levels were elevated in the 48- and 72-h cohorts; in contrast the levels in the 24-h cohort were decreased (see Table 4). Overall the changes in the 48- and 72-h cohorts differed from the changes in the 24-h cohort (*p* = 0.037), depicted in Additional file 1: Figure S2. Serum pre-albumin levels did not change consistently with fasting based on the 10 compliant patients who had data available.

**Table 4:** Baseline levels and median changes in insulin, glucose, IGF1/IGFBP1

Biomarker cohort	Baseline	Median % change after 1^st^ fast	Median % change after 2^nd^ fast	*P* value
Insulin				0.35*, 0.50**
24 h (*n* = 4)	6.95 (2.61, 18.55)	−56 % (−88 %, 65 %)	−48 % (−92 %, 242 %)	
48 h (*n* = 6)	4.86 (2.08, 11.38)	−27 % (−77 %, 131 %)	92 % (−57 %, 747 %)	
72 h (*n* = 7)	8.30 (4.15, 16.61)	−42 % (−73 %, 49 %)	−55 % (−85 %, 37 %)	
Glucose				0.13*, 0.74**
24 h (*n* = 4)	95.9 (83.8, 109.7)	3 % (−12 %, 22 %)	13 % (−4 %, 33 %)	
48 h (*n* = 6)	92.2 (82.6, 102.9)	14 % (−1 %, 30 %)	13 % (−4 %, 32 %)	
72 h (*n* = 7)	98.4 (88.9, 109.0)	−3 % (−14 %, 9 %)	−6 % (−18 %, 9 %)	
IGF-1 (ng/mL)				0.32*, 0.28**
24 h (*n* = 4)	242 (185, 315)	−30 % (−44 %, −12 %)	−31 % (−45 %, −13 %)	
48 h (*n* = 5)	177 (139, 225)	−33 % (−45 %, −18 %)	−20 % (−37 %, 1 %)	
72 h (*n* = 5)	163 (128, 207)	−8 % (−24 %, 13 %)	16 % (−5 %, −42 %)	
IGFBP1 (ng/mL)				0.09*, 0.61v
24 h (*n* = 4)	8.9 (5.7, 14)	23 % (−19 %, 87 %)	63 % (7 %, 147 %)	
48 h (*n* = 5)	8.8 (5.9, 13)	10 % (−24 %, 60 %)	5 % (−32 %, 63 %)	
72 h (*n* = 5)	3.8 (2.6, 5.7)	117 % (49 %, 215 %)	74 % (20 %, 153 %)	
B-hydroxybutyrate				0.12*, 0.037**
24 h (*n* = 4)	0.21 (0.13, 0.36)	−16 % (−48 %, 37 %)	−13 % (−47 %, 49 %)	
48 h (*n* = 5)	0.14 (0.09, 0.23)	272 % (140 %, 470 %)	82 % (9 %, 204 %)	
72 h (*n* = 5)	0.12 (0.08, 0.19)	181 % (81 %, 334 %)	24 % (−20 %, 92 %)	

These are presented only for self-reported compliant patients, and only for those with pre- and post-fasting samples available, separated by fasting duration cohort. The post-fasting blood draws were taken after 24 h of fasting in the 24 h cohort, and after 48 h of fasting in both the 48 and 72 h cohorts, and were done prior to any premedications or chemotherapy

*p-value comparing changes in levels from baseline, between the 3 fasting cohorts

**p-value comparing changes from baseline between the 24 h cohort compared to the 48 + 72 h cohorts combined

**Fig. 3:**
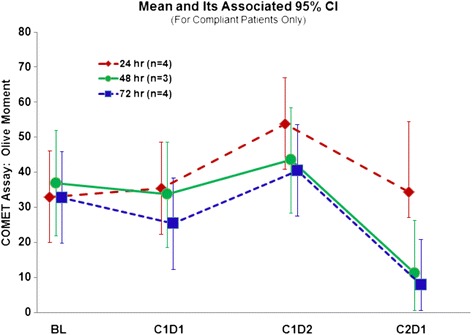
Olive moments, indicating DNA damage in peripheral blood mononuclear cells, are shown here by cohort, comparing the chemotherapy-free baseline (BL) to a sample taken after fasting, but before chemotherapy (C1D1 = cycle 1, day 1) and 24 hours after chemotherapy (C1D2 = cycle 1, day 2) and again after fasting but before chemotherapy (C2D1 = cycle 2, day 1). The difference comparing the 48 + 72 hour cohorts to the 24 hour cohort is p=0.08 by F test with ANOVA

DNA damage in normal cells was measured using peripheral blood mononuclear cells from study subjects using the COMET assay (Fig. 2). DNA damage increased in all cohorts after chemotherapy, however after fasting 48 or 72 h there was a decrease in Olive tail moment whereas the 24 h cohort continued to have evidence of increased DNA damage. At baseline the Olive tail moments, based on the COMET assay, were similar for the 3 fasting cohorts (see Fig. 3) and were essentially unchanged just after chemotherapy following the first fast (*p* = 0.61 based on least significant difference method of pairwise comparisons following overall *p*-value for changes over time *p* < 0.001). 24 h later the peripheral blood mononuclear cell DNA damage had increased slightly for all three groups (*p* = 0.042). In contrast, by the second course, the DNA damage decreased for the 48-h and the 72-h cohorts (range 0.9 to 20.7 – all values less than baseline), but not for the 24-h cohort (*p* = 0.08).

## Discussion

Calorie restriction has been associated with an increase in longevity in animals [16], and with lower rates of developing aging-related diseases such as spontaneous cancer formation [16, 17]. However, calorie restriction requires long periods to be effective and can cause a number of side effects which includes impaired immune function and wound healing. In contrast, brief cycles of STS lead to profound changes in gene expression and cellular metabolism that render normal cells more resistant to oxidative stress [6, 18]. There has been interest in studying the effect of calorie intake and nutritional composition of diet on the risk of disease progression or recurrence in patients with cancer diagnoses [19, 20]. Fasting has been studied as an adjunct to traditional therapy in rheumatoid arthritis [21] and as a means of preventing chronic disease [22, 23]. However, the use of STS to reduce chemotherapy side effects in cancer patients has, to our best knowledge, never been studied in a clinical trial. We present the first dose-escalation study of fasting in human cancer patients receiving chemotherapy, and found that up to 72 h of calorie intake <200 kcal per 24 h was safe and feasible in a population of men and women aged 31 to 75. There were no grade 3 toxicities attributed to fasting, and only one subject failed to regain any of the fasting associated weight prior to the next chemotherapy cycle. We did not find any evidence of malnutrition, although we were unable to obtain pre-albumin data in all subjects. Importantly, the safety of fasting prior to chemotherapy can only be extrapolated to a selected population of oncology patients, as we excluded those with >10 % recent weight loss, body mass index <20.5, or diabetes mellitus.

Although the study was not designed to compare the toxicity experience between cohorts, we did plan to look at changes in fasting biomarkers as part of the determination of which duration of fasting would be optimal to pursue in the randomized portion of the trial. IGF-1 has been shown to be one of the major growth factors that promote cell proliferation and growth. In model organism, inhibition of the IGF-1 signaling is associated with enhanced cellular protection against various stresses including toxins. By contrast, most tumor cells harbor oncogenic mutations in the IGF-1 signaling pathway. Constitutive activation of IGF-1 downstream mediators renders the tumor cell irresponsive to fasting-induced cellular protection. IGF-1 is significantly reduced by fasting [24], while its binding proteins exhibit divergent patterns of change in response to fasting. IGFBP-1 increases rapidly even with overnight fasting and is quickly suppressed by calorie intake [25]. The IGF-1 axis has been implicated in conferring differential stress resistance [5, 18]. Chemotherapy toxicity was found to be dramatically reduced using the liver IGF-1 deficient (LID) mouse model, which has a tissue-specific knockdown of IGF-1 resulting in approximately 70 % lower circulating IGF-1 levels than in normal mice [18]. In our subjects, there was some significant reduction of IGF-1 after fasting, which continued at the 24 h post-chemotherapy point, despite resuming normal diet. Although we would have expected greater reductions in IGF-1 in the cohorts with longer durations of fasting, the degree of change was similar between the 24 h fasting and longer duration fasting groups. The variability in insulin and IGF-1 changes likely reflects some non-compliance, perhaps a greater degree in the cohorts with longer fasting. Furthermore, values during the second fasting cycle may have been affected by dexamethasone administration with the previous cycle of chemotherapy. Since many patients joined the study after having completed one cycle of chemotherapy, even the first fasting cycle results could have been impacted.

Because there was no control group eating a regular diet, we cannot yet address the hypothesis of whether patients experience fewer or less severe chemotherapy side effects after fasting. However, we found preliminary evidence of reduced DNA-damage evident in host leukocytes after chemotherapy exposure for subjects who fasted for 72 h compared to 24 h. This is one mechanism by which we hypothesize fasting may not only reduce toxicity to normal tissues, but improve cancer treatment efficacy: by reducing damage to hematopoietic precursors and promoting hematopoietic stem cell-dependent regeneration [26, 27], blood counts might be maintained at higher levels, which would allow patients to receive their chemotherapy at full dose, on time. The reduced DNA damage in peripheral blood mononuclear cells seen based on the COMET assay results (Fig. 3) may translate into clinical benefit if host tissues, such as hematopoietic precursor cells, are protected and regenerated. Our findings in the study population mirror what we have observed in mice. We previously reported preliminary evidence of hematopoietic protection from this patient population, with less depletion of lymphocyte counts noted after repeated cycles of chemotherapy in patients who fasted for 72 h compared to those who fasted 24 h [26, 27]. Now we present additional evidence for protection against myelosuppression (Table 3), with fewer patients experiencing grade 3 or 4 neutropenia in the 48 and 72-h fasting cohorts, as well as lower rates of grade 1 and 2 thrombocytopenia. This, of course, is confounded by the varying chemotherapy regimens, and specifically by the fact that there was more gemcitabine/cisplatin in the 24 h cohort (4/6) compared to the 48- and 72-h cohorts (1/7 and 2/7, respectively). Furthermore, patients did not always enroll in the clinical trial during their first cycle of chemotherapy, so that the number of cycles an individual had experienced at the time point blood was drawn was not uniform. Nevertheless, the trends are favorable, and the lower rate of neuropathy in the 48 and 72 h fasting cohorts is particularly intriguing, given the greater number of taxane-containing regimens in these cohorts. Additional data from the ongoing randomized phase II portion of this trial will provide direct comparative data to evaluate whether STS indeed protects cancer patients against chemotherapy toxicity.

One of the reasons anti-oxidants and similar compounds have not been met with enthusiasm by the oncology community as potential methods via which to reduce chemotherapy toxicity is the concern for reducing chemotherapy efficacy. Thus, while evaluating treatment response was not feasible as an endpoint for this study due to the heterogeneity of cancer types and chemotherapy regimens as well as the inclusion of patients receiving treatment in the adjuvant setting, we felt it was important to at least evaluate whether we saw a lack of response to chemotherapy. Our observation of pathologic complete responses and radiographic responses provides reassuring preliminary evidence that the anti-neoplastic effect of chemotherapy was not negatively affected by fasting, in agreement with results published in animal studies. Our ongoing phase II trial will focus on patients being treated in the metastatic or neoadjuvant setting, and will have larger numbers of patients receiving the same chemotherapy regimens so that we can better speak to this concern, and evaluate whether enhancement of chemotherapy effect occurs.

Limitations of our study include the possibility of incomplete compliance and the variability of the composition of the “rescue” food consumed. Despite our encouragement to study subjects to honestly disclose all food and beverage consumed during the fasting period, the lack of consistent changes in glucose or ketone generation may indicate that more than 200 kcal/24 h period were consumed even in subjects whose food diaries reported <200 kcal/24 h. Furthermore, the protein content and derivation of rescue foods could theoretically influence the perception by host tissues of lack of nutrients in the environment, which may have contributed to the variable results. In the randomized trial which is currently underway, we will perform intention-to-treat analysis, but will also analyze a subgroup of “biochemically compliant” subjects to determine whether achievement of the protective fasting state, not just the attempt to achieve a fasting state, is associated with reduced chemotherapy toxicity. In addition, a separate study is underway which employs a specific fasting-mimic diet, in which subjects are provided all the food they should consume during the “fasting” period around chemotherapy (NCT01802346). This approach will eliminate the variability in the composition of the rescue food consumption and may be more acceptable to patients than aiming for zero calorie intake.

## Conclusions

In conclusion, fasting for up to 72 h, divided as 48 h before and 24 h after chemotherapy infusion, is safe and feasible in human cancer patients receiving platinum combination chemotherapy. Preliminary evidence from correlative studies supports the hypothesis that fasting may confer some protection to host tissues against chemotherapy damage to normal tissues.

## Abbreviations

COMET, gel electrophoresis method for measuring DNA strand breaks in cells; CTCAE, common terminology criteria for adverse events; DNA, deoxyribonucleic acid; ELISA, Enzyme-linked immunosorbent assay; Etc., etcetera; Ex, example; GCSF, granulocyte colony stimulating factor; IGF-1, insulin-like growth factor-1; IGFBP, insulin-like growth factor binding protein; kCal, kilocalorie; mTOR, mammalian target of rapamycin; STS, short term starvation; USC, University of Southern California

## Additional files

Additional file 1: Figure S1. Changes in IGF-1 represented as absolute changes (left side) and percentage change from baseline (right side) for the 24, 48, and 72 hour cohorts. **Figure S2.** Changes in beta-hydroxybutyrate (ketone) levels in fasting subjects, shown as absolute levels (left column) by cohort, and % changes (right column). (DOCX 182 KB)
