# Supplementary material for: Safety and feasibility of fasting in combination with platinum-based chemotherapy
Source: BMC Cancer. 2016 Jun 10;16:360. doi: 10.1186/s12885-016-2370-6 (PMC4901417; doi:10.1186/s12885-016-2370-6)

Figure S1. Changes in IGF-1 represented as absolute changes (left side) and percentage change from baseline (right side) for the 24, 48, and 72 hour cohorts.


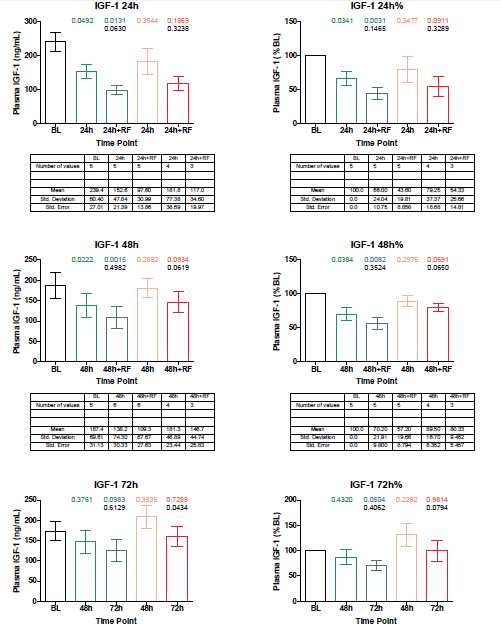


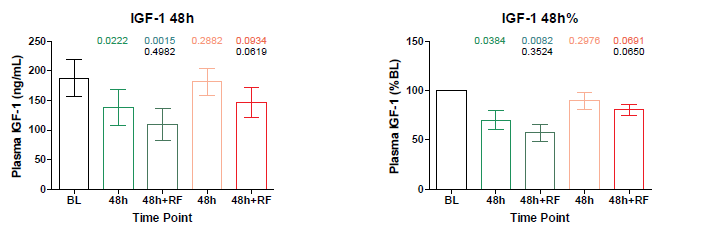


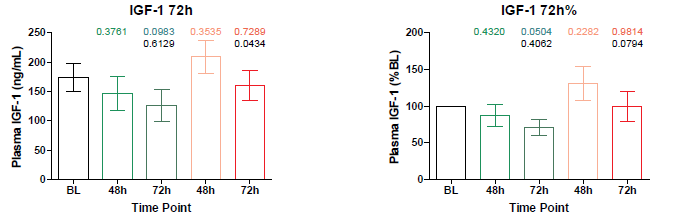


Figure S2. Changes in beta-hydroxybutyrate (ketone) levels in fasting subjects, shown as absolute levels (left column) by cohort, and % changes (right column)


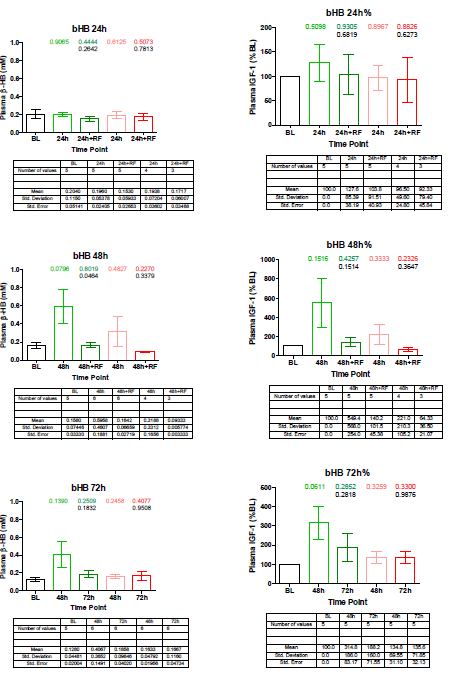

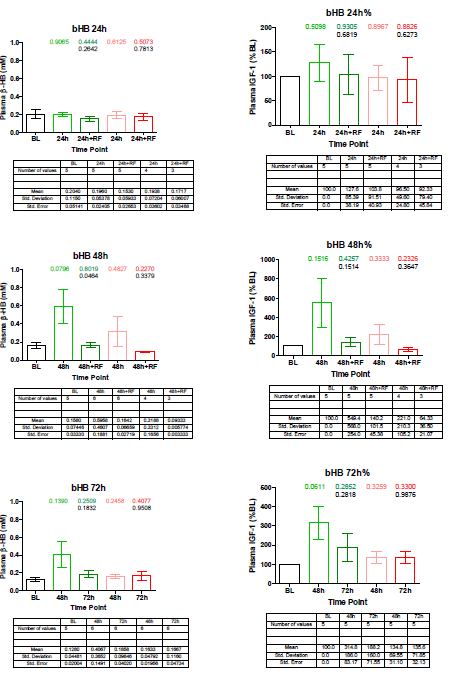

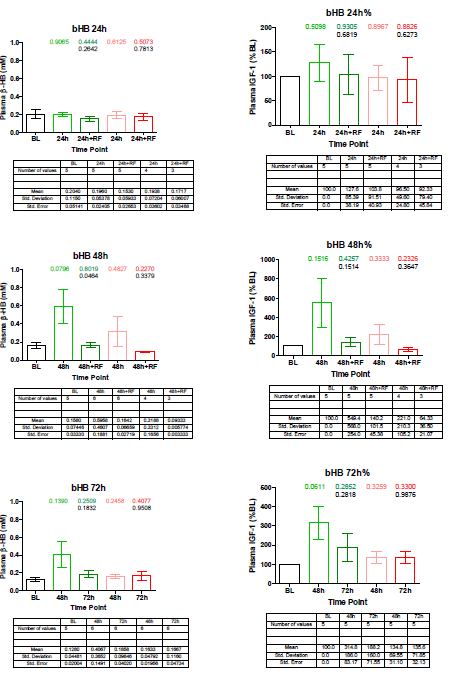

Supplement: Additional file 1: Figure S1. — Changes in IGF-1 represented as absolute changes (left side) and percentage change from baseline (right side) for the 24, 48, and 72 hour cohorts. Figure S2. Changes in beta-hydroxybutyrate (ketone) levels in fasting subjects, shown as absolute levels (left column) by cohort, and % changes (right column). (DOCX 182 KB) [file 12885_2016_2370_MOESM1_ESM.docx]
